# Aspirational autonomy and psychedelic-assisted therapy in advanced cancer: ethical lessons from clinical cases

**DOI:** 10.1093/oncolo/oyag402

**Published:** 2026-10-15

**Authors:** Keenan Wills Davis, Michelle C Bach, JoEllen Schimmels, Ali John Zarrabi

**Affiliations:** Emory University School of Medicine, Emory University, Atlanta, GA 30322, United States; Emory Center for Psychedelics and Spirituality, Emory University, Atlanta, GA 30322, United States; Emory Center for Psychedelics and Spirituality, Emory University, Atlanta, GA 30322, United States; Department of Psychiatry and Behavioral Sciences, Emory University School of Medicine, Emory University, Atlanta, GA 30322, United States; Emory Center for Ethics, Emory University, Atlanta, GA 30322, United States; Nell Hodgson Woodruff School of Nursing, Emory University, Atlanta, GA 30322, United States; Emory Center for Psychedelics and Spirituality, Emory University, Atlanta, GA 30322, United States; Department of Psychiatry and Behavioral Sciences, Emory University School of Medicine, Emory University, Atlanta, GA 30322, United States; Division of Palliative Medicine, Emory University School of Medicine, Emory University, Atlanta, GA 30322, United States; Department of Family and Preventive Medicine, Emory University School of Medicine, Emory University, Atlanta, GA 30322, United States

**Keywords:** psychedelic-assisted therapy, end of life care, palliative oncology, informed consent, ethics

## Abstract

Psychedelic-assisted therapy (PAT) is increasingly discussed as a potential intervention for anxiety, depression, and existential distress among patients with advanced cancer. Yet the ethical challenges of PAT for patients approaching the end of life remain underdeveloped, particularly for oncologists who may be asked to counsel patients or consider referral to clinical trials. This article uses 3 deidentified clinical vignettes from a clinical trial of psilocybin-assisted therapy in a palliative oncology context to examine what it means for PAT to “work.” We argue that PAT should not be evaluated only by symptom reduction or the production of mystical experiences, but also by whether it helps patients move toward valued forms of self-understanding, connection, courage, acceptance, or reconciliation. Drawing on the concept of “aspirational autonomy,” we propose that meaningful consent to PAT need not depend on full foreknowledge of an unpredictable experience. Rather, consent may be understood as a patient’s endorsement of a supported process of moral and existential aspiration. We then identify practical ethical challenges for oncology, including expectation management, therapeutic ambiguity, somatic amplification, limited time for integration, and family dynamics. We conclude by offering candidate-selection considerations for oncologists, emphasizing aspirational clarity, cognitive and psychological stability, temporal feasibility, relational support, and realistic expectations.

Implications for practice As psychedelic-assisted therapy becomes more visible to patients with advanced cancer, oncologists will increasingly be asked whether these interventions might help with anxiety, depression, or existential distress. This article offers a practical ethical framework for counseling and referral. Clinicians should distinguish patients’ aspirations from unrealistic expectations of cure, prepare patients for unpredictable and potentially challenging experiences, assess whether adequate time and support exist for meaning making, and consider whether the intervention aligns with the patient’s values, prognosis, psychological stability, and relational context.

## Introduction

In recent years, psychedelic-assisted therapy (PAT) has reentered the clinical imagination with remarkable force. For many patients with advanced cancer approaching the end of life, these treatments have come to embody a new form of hope: for equanimity, spiritual clarity, or the possibility of a less fearful relationship with their own dying.^1^^,^^2^ The scientific literature is still developing, but the stories and testimonies of patients circulate widely, carrying a moral weight that clinicians cannot ignore. Small trials of PAT in patients with cancer or other life-threatening illness have reported rapid and sometimes sustained reductions in anxiety and depression, with some participants also describing greater meaning, spiritual well-being, or acceptance.^3–6^ Yet the evidence base remains preliminary. A recent Cochrane review judged the evidence for reductions in anxiety, depression, and existential distress to be of low certainty, reflecting small samples, heterogeneous interventions, and challenges with blinding.^7^^,^^8^ It is in this context that clinicians may now find themselves fielding questions about psilocybin and related compounds. This article is written primarily for oncologists who may be asked to advise patients about PAT or referral to clinical trials.

Much of the public enthusiasm for psychedelics has been shaped by a particular narrative—one popularized in memoirs, documentaries, and a burgeoning therapeutic subculture. This is the narrative of revelation: mystical insight, relief from existential dread, and transformation that arrives in a single session. It is a powerful story, and sometimes true. Yet it can obscure the ethical complexity of PAT, especially at the end of life.^9^^,^^10^

Firstly, PAT refers to more than administration of a pharmacologic agent. Clinical protocols vary but generally include medical and psychiatric screening, preparatory sessions, a supervised dosing session with trained facilitators and physiological monitoring, and further psychotherapy-based “integration” intended to help the participant interpret and incorporate insights from the experience.^11–13^ Recent scholarship has begun to address ethical issues including responsible access, eligibility, professional training, and adaptations required when PAT is offered to people with limited prognoses.^14^^,^^15^

Additional complexity arises from the nature of psychedelic experience itself, which is often unpredictable and ineffable. Clinicians cannot yet forecast the phenomenological content of any psychedelic experience. In the language of philosophers such as L.A. Paul, psychedelic states can be “transformative experiences”: events that change what a person values, how they interpret their world, and who they take themselves to be.^16^ Patients sometimes describe ego dissolution, overwhelming emotional intensity, moments of grace or terror, and radical reorganizations of time, memory, or selfhood.^17^ Some scholars worry that this might pose a challenge to conventional models of informed consent.^18^ However, uncertainty and identity-altering consequences are not unique to PAT. Cancer surgery and chemotherapy can also result in changes to a person’s values, priorities, and how they experience the world. Ordinary informed consent requires adequate disclosure, understanding, voluntariness, capacity, and authorization under conditions of acknowledged uncertainty.^19^ Thus we argue that PAT intensifies a familiar problem of medical decision-making rather than rendering conventional consent impossible.

Rather than seeing challenges to informed consent as a barrier to treatment, we suggest they invite a richer ethical framework, grounded in aspiration. Philosopher Agnes Callard has argued that people often choose experiences they cannot fully understand in advance because they aspire to become someone slightly different: a person who can face loss with more courage, speak to loved ones with greater honesty, or accept the finitude of life with less bitterness.^20^ The concept of “aspirational autonomy” can supplement ordinary informed consent by asking whether an unpredictable intervention plausibly serves a direction of becoming that the patient can presently endorse.^21^ This model can help individuals think through experiences whose value may become intelligible only through participation.

For patients approaching the end of life, aspiration takes on a particularly poignant quality. Time is short; functional capacity is fragile; the emotional lives of families are strained. Integration—the process for making meaning of psychedelic experiences—may be compressed into days or weeks rather than months.^12^^,^^15^ Yet aspiration does not disappear simply because the horizon narrows. Rather, it is often in these final stretches of life that aspiration may become clearest.^22^^,^^23^

This article explores ethical complexities that arise from PAT for cancer patients through the stories of 3 deidentified participants in an open-label feasibility study of psilocybin combined with palliative care for cancer patients experiencing demoralization and chronic pain (NCT05506982).^24^^,^^25^ Identifying details have been altered to protect patient confidentiality. Each case illustrates a distinctive ethical experience that can occur when PAT enters the world of oncology. Through these cases, we examine what it means for PAT to “work” in end of life care, how to conceptualize consent under conditions of uncertainty, how to manage expectations, and how clinicians might discern which patients are appropriate for PAT referral. Our aim is not to advocate for or against these treatments, but to illuminate the ethical terrain and show how patients’ experiences complicate simple narratives of promise or harm.

## Three vignettes

### Patient 1

A 39-year-old woman with advanced ovarian cancer and cancer-related pain on chronic opioid therapy enrolled in the clinical trial. Holding the capsule in her palm, she told her therapy team, “We can do hard things,” and swallowed the medication.

In reflections after the session, she wrote, “I felt warm, happy. A happiness that I haven’t felt in a long time.” She experienced vivid imagery and a sense of traveling through “different dimensions.” When fear arose, she reported that the medicine seemed to calm her and remind her she was in control. She described feelings of gratitude, insight, and connection, adding that her fear of death receded during the session and her pain had subsided. She concluded: “Before this experience, I felt hopeless regarding my pain. I felt that cancer took away my happiness. This showed me I’m still the same person. I’m still whole. Cancer doesn’t define me. I feel replenished, recharged.”

In the weeks following her session, her cancer-related pain gradually returned. She remained on opioid therapy but described a “changed relationship with oxycodone,” explaining that she now felt better able to distinguish when she needed medication for physical pain from occasions when she had previously used it to soothe anxiety or existential distress. She told the study team that the experience was “exactly what I needed.”

### Patient 2

A 56-year-old man with multiple myeloma and painful lytic lesions described feeling that he had “lost everything” since his cancer diagnosis, noting that depression had strained his relationships with his family. During preparation sessions, he expressed hope that the intervention would help him feel “mentally and emotionally better with less pain and more controlled thoughts.”

As his disease progressed, however, his expectations shifted. He began telling the team that he hoped psilocybin might directly treat his tumors or provide analgesia. Despite repeated clarification that the intervention was not intended to treat cancer itself, he continued to frame it as a potential cancer treatment. The study team ultimately determined that he was not an appropriate candidate for dosing because his goals for participation had become anchored in the belief that the drug could alter the course of his cancer.

### Patient 3

A 60-year-old woman with advanced lung cancer enrolled in the trial hoping to have a mystical experience before choosing hospice care over continued cancer-directed therapy. Her dosing day proved physically and emotionally challenging. She experienced nausea and pain and described sensations resembling labor contractions. She later wrote that she was “devastatingly disappointed.”

Later in the session, however, she described a vision of her soul confined within a narrow space and looking outward toward a clearing. In that moment she realized she did not feel ready to die. She repeated to herself that in her exhaustion she was “weary but joyful.”

During subsequent integration sessions, she reported that she had decided against hospice and planned to continue cancer treatment. She also reflected that she had already experienced profound mystical moments in her life and did not need to pursue another one. She ultimately reported little desire to repeat PAT.

## Psychedelic-assisted therapy in an aspirational autonomy framework

These vignettes resist a single definition of success. One way to conceptualize these outcomes is through the abovementioned lens of “aspirational autonomy.” PAT is effective not when it produces bliss or mystical unity, but when it helps patients move closer to the kind of person they wish to be in the time they have left. For Patient 1, this meant cultivating equanimity that allowed her to remain emotionally present in the face of ongoing pain and to relate to her illness without feeling wholly defined by it. For Patient 3, it meant confronting disappointment and physical suffering in a way that clarified her desire to continue living and affirmed her resilience. More than merely affective, this type of alignment concerns what matters most to patients. Such moral attunement is not simply reducible to changes on a symptom scale.^23^^,^^26^

This also helps explain why challenging or ambivalent experiences can nonetheless be effective.^27^^,^^28^ In palliative care, clinicians often witness how difficult emotions can, when supported and understood, lead to deeper conversations and connection. Psychedelic experiences, with their capacity to unsettle the habitual defenses that patients develop over years of illness, can sometimes catalyze this process. What may matter most is not whether the experience is comforting in the moment, but whether it serves the patient’s aspirational trajectory. In this sense, PAT at the end of life can be understood as a form of moral accompaniment, supporting patients to face suffering, express fears and hopes, and approach death with greater intention.^22^^,^^23^

Practically, then, how should clinicians best support patients’ autonomy under this consent framework grounded in aspiration? Traditional informed consent assumes that clinicians can outline risks and benefits of treatment in advance with sufficient clarity that patients can make rational choices in light of their values. But when the experience itself is unforeseeable and potentially value-altering, this assumption seems to break down. All medical treatments involve uncertain benefits and harms, and many also have consequences that patients cannot fully imagine in advance; PAT is therefore not categorically outside the structure of informed consent.^29^ Its ethical distinctiveness is ultimately one of degree and configuration: the acute phenomenology is especially difficult to anticipate; patients may assign unusual authority to perceived insights; and changes in beliefs, values, or identity may be understood as part of the desired benefit.^18^^,^^30–32^ These features warrant enhanced forms of informed consent, supplementing established standards.^30^^,^^31^ In addition to addressing known and uncertain benefits, burdens, risks, and alternatives, counseling grounded in aspirational autonomy adds a further fundamental question: What direction of change does the patient hope this process might serve? In this view, the cancer patient who pursues PAT would not be consenting to predicted phenomenology—such as a mystical experience—but to a supported, iterative, developmental process of moral formation: toward courage, acceptance, reconciliation, or spiritual clarity, for example.

Consider Patient 1, whose aspiration was to remain emotionally present in the face of suffering rather than retreat into fear or despair. Her choice to “do hard things” was an act of self-formation under conditions of radical uncertainty. She could not know what would arise during the session or whether relief from pain would persist. What she endorsed instead was the possibility of reorienting herself toward illness with greater equanimity. The primary significance of the experience lay not in the temporary reduction of pain but in the shift it enabled in her relationship to that pain and to the illness that had come to dominate her life.

The contrast with Patient 2 is instructive. His aspiration was not existential but biomedical. He sought a cure. His choice, while understandable, was organized around a false belief about the nature of the treatment.^33^ No amount of aspirational framing can transmute empirical error into autonomy. His case shows the outer boundary of ethical practice in psychedelic medicine. A patient may legitimately hope for cure, analgesia, clarity, or reconciliation, but meaningful authorization requires understanding whether the proposed intervention is reasonably capable of serving that hope.

The end of life imposes a further complication. Our aspirations typically unfold over long durations. For instance, one becomes a philosopher or an artist over many years. But dying compresses the available timescale. Patients approaching the end face a foreshortened horizon within which to pursue personal change. Yet the human capacity for aspiration does not vanish. A patient might aspire to express love they had long withheld, or to forgive a family member, or to face death without avoidance. We might call this “finite-horizon aspiration”—aspiration under severe temporal constraint.^15^^,^^22^ PAT may catalyze this kind of work, but only when the patient understands that the aim is moral rather than curative, and only when they have the support and time to integrate what emerges.^12^^,^^23^

Consent for PAT, then, should not be framed as an agreement to a known experience, but as a commitment to a supported journey whose content cannot be fully specified in advance. The guiding questions in conversation with patients become: What kind of person do you hope to be in the time that remains? And are you willing to face what may arise if doing so might help you move toward that aspiration? This reframing centers the patient’s local moral world rather than the phenomenology of the drug. It situates consent as a relational and narrative process, not a formal and transactional event. And it aligns PAT with the fundamental practice of palliative care: helping patients articulate what matters most when time is short.^22^^,^^34^

Patient 3’s case is the most ethically difficult of the 3, and, for that reason, perhaps the most revealing of both the potential of PAT and the value of an aspirational autonomy framework. Instead of her hoped-for mystical experience, she encountered intense discomfort and emotional disappointment that felt initially like failure. Through post-dosing integration with specialized PAT teams, she came to see that the deeper aspiration beneath her desire for transcendence was not merely escape from suffering but clarity about how she wished to live. This experience precipitated a consequential reversal: from planning hospice enrollment to pursuing further cancer-directed treatment. Precisely because the decision was so consequential, the apparent insight could not simply be accepted at face value.

Psychedelic integration literature and some clinical programs advise patients to avoid major life changes for several weeks after dosing, partly because feelings of certainty or insight can be misleading.^28^^,^^32^ Yet this caution should not become an inflexible prohibition, especially in the context of advanced illness. The ethical status of a post-psychedelic decision depends on at least 3 distinctions. First, a patient should not make consequential decisions while acutely intoxicated. Once acute drug effects resolve, however, the question is no longer simply whether the patient has taken a psychedelic, but whether the patient has decision-making capacity for the choice at hand.^35^ Second, many major life decisions can be deferred, whereas cancer treatment decisions may be time-sensitive and delay may itself cause harm. Third, a psychedelic experience may disclose value-laden information, such as “I am not ready to die,” but it cannot establish the appropriateness of a specific medical treatment.

Patient 3’s experience should therefore be interpreted as prompting reconsideration rather than independently justifying continued treatment. Her realization that she wished to live was ethically significant, but it still required additional counseling across more than one conversation: consideration of the likely benefits and burdens of treatment, assessment of decisional capacity, and attention to whether her preference persisted and remained consonant with her broader values. This is what makes the case so instructive. Aspirational autonomy provides a framework for taking those insights seriously while subjecting them to the relational, clinical, and temporal safeguards necessary for responsible action. Such safeguards preserve decisional caution without imposing a waiting period that could itself be harmful in end of life settings.^15^ In the end, PAT helped bring Patient 3’s deepest aspirations into view and reopen a question about how she wished to live. The aspirational autonomy framework in turn helps determine how those aspirations should responsibly shape what comes next.

## Practical ethical challenges in oncology

If this aspirational model captures the ethical structure of PAT, the practical challenge becomes how to obtain meaningful consent within the clinical realities of oncology. Consent at the end of life is always fragile, shaped by urgency, fear, family dynamics, and the pressures of medical decision-making. PAT introduces new complexities.

### The burden of expectation

Public enthusiasm has popularized a narrative of psychedelic experience as reliably mystical, luminous, and liberating.^9^^,^^10^ Patients may therefore expect a profound breakthrough or moment of transcendence. When the actual experience is confusing, painful, or even uneventful, they may feel betrayed or blame themselves.^27^^,^^28^

Clinicians may find it helpful to include explicit language such as:

- Some experiences are peaceful; others are confusing or unsettling.
- A difficult experience does not mean the treatment has failed.
- Meaning often emerges through later reflection and integration.

This kind of language grounds the patient in the purpose and reality of the treatment, countering the cultural scripts that promise revelation.

### Therapeutic ambiguity

The oncologic setting also amplifies the risk of misunderstanding. Patients may project biomedical hopes onto PAT, as did Patient 2. Clinicians must distinguish between the desire for cure and the aspiration for clarity. Without this distinction, consent cannot be valid. Furthermore, because expectations can shift as illness progresses, clarifications may need to be repeated across multiple conversations.

Transparency is essential:

- There is no evidence that this treatment will shrink tumors.
- Its goal is to help you live with your illness, not to change the illness itself.
- It is not a substitute for cancer-directed therapy.

These statements must be delivered with empathy, acknowledging the emotional logic behind the misconception while gently correcting the factual error.

### Somatic amplification

PAT introduces physiological dynamics that are particularly relevant for oncology patients. Psychedelics can function as “nonspecific amplifiers” of not only mental phenomena but also bodily sensations, including pain.^15^^,^^24^^,^^36^^,^^37^ By amplifying somatic sensation, patients may be drawn into confrontation with suffering. Though palliation and transcendence of pain are possible with PAT, they may be experienced with and sometimes through this confrontation.

Preparation sessions must address this possibility:

- The medication is an amplifier, not an anesthetic.
- You may feel physical symptoms more acutely, not less.
- The experience may be an immersion in the body, not an escape from it.
- When done in a medical setting, your provider can treat discomfort that arises during psychedelic dosing.

By describing the body as a potential site of confrontation, the clinician better inoculates the patient against the shock of unexpected pain, reframing it as a known, if difficult, part of the territory.

### Limited integration time

As discussed, integration is central to PAT. Without it, the experience may remain fragmented, overwhelming, or unproductive. Yet in the context of advanced illness, time may become short. Cognitive decline, physical deterioration, or escalating symptoms may limit the patient’s ability to reflect and articulate.

Clinicians must ensure that:

- there is enough time for at least minimal integration
- the palliative care team is prepared to support ongoing reflection
- family members can help support the meaning of the experience

When integration is not realistically possible, due to rapid decline or profound instability, PAT may not be appropriate.

### Family dynamics

Lastly, psychedelic experiences often ripple outward.^14^^,^^15^ A patient’s newfound sense of acceptance may comfort some family members while leaving others feeling prematurely confronted with loss. A profound experience may expose unresolved conflicts. The treatment team must be attentive to the relational “matrix” into which the experience will be integrated.^38^ Families also require support and guidance.

These practical challenges underscore the importance of thoughtful preparation, clear communication, and ethical discernment when introducing these experiences into the fragile moral landscape of advanced illness.

## Who is an appropriate candidate?

To assist oncologists navigating these questions, we propose a heuristic grounded in palliative care and aspirational autonomy. The following considerations may help clinicians determine whether PAT is appropriate for a particular patient.

### Adequate understanding and voluntariness

The patient should understand the experimental status and purpose of the intervention, its structured therapeutic components, the possibility of difficult or disappointing experiences, available alternatives, and material risks. Consent should be treated as an ongoing conversation rather than a single event. Patients should understand that they can decline or withdraw and that asking about psychedelics will not compromise their oncology or palliative care.

### Aspirational clarity

The patient should be able to articulate, at least roughly, concerns or goals that are reasonably connected to the intervention: reducing fear, reconnecting with family, approaching illness more openly, exploring questions of meaning, or becoming better able to live with uncertainty. They need not specify experiential details, but they should have a sense of direction: toward acceptance, connection, courage, etc.

### Cognitive and psychological stability

The patient must have enough clarity to understand the aim of treatment and the unpredictability of the experience. Delirium, psychosis, or extreme emotional fragility would preclude safe participation.

### Temporal feasibility

There must be adequate time to conduct preparation sessions, complete the dosing session, and support integration. Patients with short prognoses may not have the time needed for this full therapeutic arc.

### Relational support

Family members, palliative care clinicians, chaplains, or psycho-oncologists should be available to support integration. PAT can reshape relationships; relational support is essential.

### Realistic expectations

Patients should not be anticipating cure, ecstatic experience, or guaranteed mystical insight. Their context-specific aspiration must be distinguished from culturally influenced fantasies.

These considerations are not validated predictors of response or a substitute for specialist screening. But they are a practical ethical heuristic for determining whether referral is plausibly aligned with the patient’s goals and clinical circumstances.

## Conclusion

PAT should be seen as a promising but still experimental process for some patients to engage the challenges of dying with greater intentionality. These therapies can unsettle, illuminate, and transform. Aspirational autonomy provides a useful framework for ethically evaluating PAT, supplementing ordinary informed consent by making explicit and proactively addressing the question of whether an intervention plausibly serves a direction of becoming that the patient can presently endorse.

Oncologists occupy an important position in this process. Through longitudinal care, oncologists often serve as narrative stewards in the lives of their patients, developing a deep understanding of how individuals interpret their illness and what they value and hope for. This knowledge can help clinicians discern when PAT aligns with a patient’s aspirations and when it may not.

PAT is one emerging tool among many within the broader work of palliative care. But like all powerful clinical tools, it must be approached with humility, discernment, and a deep attunement to the moral worlds of patients and families. As PAT moves from experimental settings toward broader clinical availability, the ethical responsibility will increasingly rest not only with psychedelic specialists but with the oncologists who help patients decide whether this path is worth walking.

## Data Availability

No new datasets were generated or analyzed for this article. The clinical vignettes are derived from previously collected clinical research data and have been deidentified and modified to protect patient confidentiality.
